# Dietary LPC-Bound *n*-3 LCPUFA Protects against Neonatal Brain Injury in Mice but Does Not Enhance Stem Cell Therapy

**DOI:** 10.3390/nu16142252

**Published:** 2024-07-12

**Authors:** Eva C. Hermans, Carlon C. E. van Gerven, Line Johnsen, Jørn E. Tungen, Cora H. Nijboer, Caroline G. M. de Theije

**Affiliations:** 1 Department for Developmental Origins of Disease, University Medical Center Utrecht Brain Center and Wilhelmina Children’s Hospital, Utrecht University, 3508 AB Utrecht, The Netherlands; 2 Aker BioMarine Human Ingredients AS, Oksenøyveien 10, 1327 Lysaker, Norway

**Keywords:** neonatal hypoxic-ischemic brain injury, Lysoveta, LPC-DHA, LPC-EPA, krill oil, oxidative stress, oral supplementation, intranasal, mesenchymal stem cell therapy, neuroprotection

## Abstract

Neonatal hypoxic-ischemic (HI) brain injury is a prominent cause of neurological morbidity, urging the development of novel therapies. Interventions with *n*-3 long-chain polyunsaturated fatty acids (*n*-3 LCPUFAs) and mesenchymal stem cells (MSCs) provide neuroprotection and neuroregeneration in neonatal HI animal models. While lysophosphatidylcholine (LPC)-bound *n*-3 LCPUFAs enhance brain incorporation, their effect on HI brain injury remains unstudied. This study investigates the efficacy of oral LPC-*n*-3 LCPUFAs from Lysoveta following neonatal HI in mice and explores potential additive effects in combination with MSC therapy. HI was induced in 9-day-old C57BL/6 mice and Lysoveta was orally supplemented for 7 subsequent days, with or without intranasal MSCs at 3 days post-HI. At 21–28 days post-HI, functional outcome was determined using cylinder rearing, novel object recognition, and open field tasks, followed by the assessment of gray (MAP2) and white (MBP) matter injury. Oral Lysoveta diminished gray and white matter injury but did not ameliorate functional deficits following HI. Lysoveta did not further enhance the therapeutic potential of MSC therapy. In vitro, Lysoveta protected SH-SY5Y neurons against oxidative stress. In conclusion, short-term oral administration of Lysoveta LPC-*n*-3 LCPUFAs provides neuroprotection against neonatal HI by mitigating oxidative stress injury but does not augment the efficacy of MSC therapy.

## 1. Introduction

Perinatal asphyxia is a major cause of hypoxic-ischemic (HI) brain injury in the new-born. In affluent countries, the occurrence of HI brain injury is estimated to range between 1.3 and 1.7 cases per 1000 live births [1]. Neonatal HI brain injury is a significant contributor to neonatal mortality and can result in various lifelong health challenges such as intellectual disabilities, epileptic seizures, and cerebral palsy [2]. These detrimental consequences result from a series of harmful processes in the brain, including oxidative stress and excitotoxicity, ultimately culminating in the death of neurons over hours to days following the initial insult [2]. As current hypothermia treatment offers only partial protection, there is an urgent need for novel therapeutic strategies to combat HI injury [3,4]. Nutritional supplementation emerges as such a novel treatment option. Notably, nutritional intervention is considered safe and has the potential to be easily integrated into clinical practice [5]. Moreover, its limited side effects make it an attractive candidate for combination with additional therapies [5,6].

The brain is particularly rich in lipids with around 30% consisting of *n*-3 LCPUFAs, such as docosahexaenoic acid (DHA) and eicosapentaenoic acid (EPA) [7]. Specifically, DHA plays a vital role in the cell membranes of gray matter [8]. By interacting with membrane proteins and influencing downstream signal transduction, DHA can influence synaptic activity, promote neurogenesis, and neuronal survival [9]. Interestingly, DHA is not confined to neurons but also has been found in membranes of astrocytes, microglia, and oligodendrocytes, allowing it to influence neuroinflammation or myelin formation [7]. Furthermore, DHA can be converted into bioactive metabolites such as oxylipins (e.g., NPD1) and synaptamides. Similar to DHA itself, these bioactive metabolites can reduce neuroinflammation and apoptosis while stimulating neurite growth and synaptogenesis [9,10].

DHA uptake in the brain is dependent on a constant supply from the blood which is influenced by the dietary intake of DHA [11]. Intriguingly, DHA transport into the brain is mainly dependent on its binding to lysophosphatidylcholine (LPC) [12]. The transporter Major Facilitator Superfamily Domain containing 2a (Mfsd2a) specifically transports LPC-esterified *n*-3-LCPUFAs across the blood–brain barrier into the brain parenchyma [12,13]. Indeed, some studies have shown that the supplementation of LPC-DHA but not of free DHA or DHA bound to triglycerides (TAG-DHA) leads to the accumulation of DHA in the brain [13,14]. In addition, only LPC-DHA leads to subsequent improvements in memory function in adult mice [13,14]. Similarly, only LPC-EPA enriches both EPA and DHA in the brain [15]. Altogether, these data suggest that nutritional supplementation containing LPC-DHA and LPC-EPA shows the most promise to yield the beneficial effects of DHA and EPA enrichment in the brain. Surprisingly, no studies have examined the impact of nutritional supplementation with LPC-DHA or LPC-EPA on HI injury in mice.

Neonatal HI brain injury has been shown to reduce the amount of DHA in the brain [16,17]. Nutritional supplementation with DHA could complement this deficiency. Furthermore, studies showed that intraperitoneal free or tri-DHA pretreatment or tri-DHA treatment shortly after HI injury in neonatal rats and mice reduced brain volume loss, glial reactivity, and microglia activation and normalized anxiety-like behavior, long-term working memory, and sensorimotor functioning [18,19,20,21]. Similarly, a long-term DHA-rich diet in HI-injured mice reduced gray and white matter lesion size, microglia activation, and glial reactivity in male mice and improved novel object recognition memory in both sexes [16]. Moreover, multiple studies showed that intraperitoneally or intravenously administered free or tri-DHA shortly after HI injury in rats or piglets reduced lipid peroxidation, preserved mitochondrial integrity, reduced mitochondrial ROS production, and improved mitochondrial Ca^2+^ buffering capacity [20,22,23].

Currently, one of the most promising therapies for neonatal brain injury is mesenchymal stem cell (MSC) therapy [24,25,26,27]. MSCs are non-immunogenic cells that can be extracted from different sources such as bone marrow [28]. Intranasal administration of MSCs has been shown to effectively reduce lesion size and improve long-term sensorimotor and cognitive outcome in experimental models for neonatal brain injury [24,25,26,29,30,31]. Upon intranasal administration, MSCs migrate specifically to the lesion site, are short-lived, and do not integrate [26]. During this short presence at the lesion site, MSCs react to the HI environment by secreting various factors that have beneficial effects on the injured brain by boosting neurogenesis and dampening neuroinflammation [24,26,32,33,34]. MSCs have a large therapeutic window being effective when administered up until 10 days post-HI and stimulate the late repair of the neonatal HI-injured brain, which may complement the early neuroprotective window of *n*-3 LCPUFAs [26,35]. Additionally, *n*-3 LCPUFAs may provide building blocks for the neuronal repair process [29], further boosting neurite outgrowth and synapse formation [9]. Indeed, Ghazale et al. showed that DHA enhances the therapeutic potential of neural stem cell treatment after traumatic brain injury, by reducing apoptosis and promoting neurogenesis [6].

The current study aims to assess the therapeutic efficacy of nutritional supplementation with LPC-DHA and LPC-EPA from Lysoveta on lesion size and functional outcome in a mouse model of neonatal HI brain injury. Potential underlying mechanisms of Lysoveta were assessed using in vitro models of neuronal injury. Lastly, the additive benefit of Lysoveta supplementation on the neuroregenerative effects of intranasal MSC therapy following neonatal HI brain injury was investigated. Together, this study provides valuable insights into the prospects of LPC-bound *n*-3 LCPUFA supplementation for infants with HI injury.

## 2. Materials and Methods

### 2.1. Animals and HI Injury Model

All procedures were carried out according to the Dutch and European international guidelines (Directive 86/609, ETS 123, Annex II) and the Central Authority for Scientific Procedures on Animals (The Hague, The Netherlands) and approved by the Experimental Animal Committee Utrecht (University Utrecht, Utrecht, Netherlands). All efforts were made to minimize suffering. This paper is written in accordance with the ARRIVE guidelines [36]. C57Bl/6 mice (OlaHsa, ENVIGO, Horst, The Netherlands) were kept in individually ventilated cages with woodchip bedding, cardboard shelters, wooden nibble stick and tissues provided, on a 12 h day/night cycle (lights on at 7:00 a.m.), in a temperature-controlled room at 20–24 °C and 45–65% humidity with ad libitum food and water access. Mice were bred in-house by placing males and females together in 1:2 ratio for 10 days. Afterwards, dams were housed solitarily to give birth. The day of birth was considered as postnatal day (P)0. Litter size was controlled between 6 and 8 pups, to ensure the adequate feeding of each pup. HI injury (Vannucci-Rice Model, [37]) was induced in 9-day-old pups by unilateral (right) carotid artery ligation under isoflurane anesthesia (5–10 min; 5% induction, 3–4% maintenance with flow O_2_:air 1:1), followed by recovery with their mother for at least 75 min and subsequently systemic hypoxia at 10% O_2_ for 45 min in a temperature-controlled humidified hypoxic incubator. Control animals (sham procedure) were subjected to anesthesia and surgical incision only and were without exposure to hypoxia. Xylocaine (#N01BB02, AstraZeneca, Cambridge, UK) and Bupivacaine (#N01BB01, Actavis, Allergan Inc., Dublin, Ireland) were applied to the wound for pre- and post-operative analgesia, respectively. Litters received a turning wheel on a colored plastic shelter as cage enrichment starting from two days post-injury. Mice were treated as described below. Group sizes were determined based on the effect size in previous experiments by performing a power analysis. The power analysis was based on an effect size of the lesion size of 1, alpha of 0.05 with Bonferroni correction for multiple comparisons, and power of 0.8, resulting in a minimum number of animals of 24 per group (G-power 3.1.9, Universität Kiel, Kiel, Germany). The number of animals used in this study is depicted in Table 1. Animals within each litter were randomly assigned to the experimental groups aiming for an equal male-to-female ratio to the most feasible extent. Mice were weaned after finishing the behavioral tests and housed in same-sex groups per litter. Mice were euthanized at P37 by overdose of 20% pentobarbital, followed by transcardial perfusion with phosphate buffered saline (PBS, #524650-1, VWR, Radnor, PA, USA) followed by 4% paraformaldehyde (PFA, #4078.9020, VWR), and brains were collected for further analysis.

### 2.2. MSC Culture

GIBCO^®^ Mouse (C57BL/6) mesenchymal stem cells (#S1502-100, Thermo Fisher Scientific, Bleiswijk, The Netherlands) were used in this study and cryopreserved at passage 9 in vials containing 1 × 10^6^ cells. Cells were thawed in MSC medium consisting of DMEM:F12 GlutaMax (#31331093, Fisher Scientific, Landsmeer, The Netherlands), 10% FCS (#10270106, Fisher Scientific), 0.05% gentamycin (#15710064, Fisher Scientific), and 1% penicillin/streptomycin (P/S, #15140163, Fisher Scientific) in T75 flasks (#353110, Corning Life Sciences, Amsterdam, The Netherlands), grown until 80% confluency at 37 °C, 5% CO_2_, and 90% humidity (MCO-19M-PE, PHCbi, Etten-Leur, The Netherlands), according to the manufacturer’s instructions, and passaged once before use.

### 2.3. Treatment: Nutritional Lysoveta Supplementation and/or MSC Administration

Immediately following systemic hypoxia, mice were orally treated with Lysoveta, a product rich in LPC-*n*-3 LCPUFA (Table 2, Aker Biomarine Human Health Ingredients AS, Lysaker, Norway), or with the vehicle solution coconut oil (#C1758, Merck KGaA, Saint Louis, MO, USA), rich in saturated short-chain fatty acids (Table 3).

Lysoveta is a supplement derived from the oil of Antarctic krill (*Euphausia superba*) through the enzymatic hydrolysis of phosphatidylcholine, resulting in high levels of LPC-DHA and LPC-EPA. To reduce viscosity and allow for oral gavage, the Lysoveta product was diluted 4× in coconut oil and administered with a sterile plastic feeding tube (22 ga, 38 mm, #FTP22-38 Instech Laboratories, Leipzich-Markkleeberg, Germany) attached to a 25 µL Hamilton syringe (Hamilton company, Reno, NV, USA) at a dose of 5 µL/g body weight (equal to 1.25 µL pure Lysoveta/g body weight) directly after hypoxia and for 7 consecutive days. This resulted in a daily dosage of 117.5 mg/kg body weight DHA and 210.63 mg/kg body weight EPA supplementation, which has been shown to be sufficient to result in increased DHA or EPA brain incorporation [13,15]. On P12, mice were intranasally treated with 0.5 × 10^6^ MSCs (Thermo Fisher Scientific) or vehicle (D-PBS, D8537, Merck KGaA) per animal by the administration of 3 rounds of 2 μL per nostril. Additionally, 30 min prior to administration, hyaluronidase (100U, #H4272, Merck KGaA) was administered intranasally to increase the permeability of the connective tissue in the nasal cavity (3 rounds of 2 µL per nostril).

### 2.4. Immunohistochemistry

Collected brains were post-fixed in 4% PFA for 24 h and dehydrated in increasing ethanol concentrations followed by embedment in paraffin. Coronal sections (8 µm) were cut at the hippocampal level at bregma level −1.70 (in adult mice). Sections were stained with anti-microtubule-associated protein 2 (MAP2) or anti-myelin basic protein (MBP) antibodies to analyze gray or white matter damage, respectively, as a primary outcome measure. All sections were deparaffinized and blocked with 3% H_2_O_2_ (#1.072.101.000, VWR) in methanol for 20 min and afterwards hydrated in decreasing ethanol concentrations. Antigen retrieval was performed for 3 min at 95 °C in 10 mM citrate buffer (pH 6.0) for MAP2 staining or for 15 min in 0.05 M TRIS HCL/0.01 M EDTA (pH: 9.0) for MBP staining followed by blocking with 5% normal horse serum (NHS, #26050088, Invitrogen, Waltham, MA, USA) in PBS or 20% normal goat serum (NGS, X090710-8, Agilent Technologies, Amstelveen, The Netherlands) in 0.025% Triton (X100, Merck KGaA) in PBS for 30 min, respectively. Afterwards, slides were incubated with 1:1000 mouse-anti-MAP2 antibody (#M4403-2 mL, Merck KgaA) in 2% NHS in PBS or 1:2000 rabbit-anti-MBP antibody (#ab218011, Abcam, Cambridge, MA, USA) in 0.025% Triton and 10% NGS in PBS overnight at 4 °C. The following day, slides were incubated with 1:100 horse-anti-mouse biotin antibody (#BA-2000, Vector Laboratories, Newark, CA, USA) in PBS or 1:100 goat-anti-rabbit biotin antibody (#BA1000, Vector Laboratories) in PBS for 45 min at RT. Next, the slides were washed in PBS and incubated for 30 min with AB complex (#PK-4000, Vector Laboratories) in PBS at RT. Slides were washed briefly in Tris-HCl (pH 7.6) and stained with DAB solution (#D5637-10G, Merck KgaA) until the desired intensity was reached (±5 min). Lastly, sections were dehydrated and embedded with DEPEX (#18243.01, Serva Electrophoresis GmbH, Heidelberg, Germany). Full-section images were captured with a Nikon D1 digital camera (Nikon, Tokyo, Japan). Area measurements (in pixel) were manually performed by a blinded observer using Adobe Photoshop CS6 or Fiji 1.53 (NIH) for MAP2- or MBP-stained brains, respectively. The positive stained area was measured in both the ipsilateral (lesioned) and contralateral (non-lesioned) hemispheres of each brain section. Ipsilateral MAP2^+^ or MBP^+^ area loss was calculated as (1 − ipsilateral positive area/contralateral positive area) × 100%, to correct for differences in brain size between animals.

### 2.5. Behavioral Testing

For behavioral experiments, the day/night cycle was reversed at P24, and behavioral tasks were performed during the dark phase under red light conditions. Behavior was scored blinded by experienced researchers. Arenas were cleaned with soapy water in between each trial. Mice that showed severe repetitive turning behavior after HI were omitted from all behavioral analyses (HI VEH: *n* = 1, HI MSCs: *n* = 3, HI Lysoveta + MSCs: *n* = 4).

#### 2.5.1. Open Field Task

Rodents naturally tend to cautiously explore the environment while avoiding open areas. Mice that express anxiety-like behavior tend to spend more time on the sides of the arena than crossing into the inner zone of the arena. The open field task was conducted on P30. Mice were recorded freely roaming in a rectangular plexiglass arena (560 × 330 × 200 mm) for 10 min. Movies were automatically analyzed by Ethovision XT software version 15 (Noldus, Wageningen, The Netherlands) by digitally determining an inner zone starting at 10 cm from the walls. Time spent in the inner zone and total distance moved were measured.

#### 2.5.2. Spatial Memory

The novel object recognition task (NORT) is used to test spatial memory and reflects hippocampal and perirhinal cortex function. The NORT was conducted on P34 as previously described [16]. Mice were habituated in a rectangular plexiglass arena (560 × 330 × 200 mm) for 10 min on four consecutive days prior to the test day. On the fifth day, mice were placed in the same arena, which now contained two identical objects; a cylinder made of four stacked blue 50 mL Falcon tube-caps (Corning). Mice were left to explore both identical objects for 10 min. Mice were then returned to the home cage for one hour. During the novel object trial, one object was replaced by a novel object, which was a yellow, 8-hole Duplo brick (The Lego Group, Billund, Denmark), and mice freely explored both objects for 10 min while being recorded by a BlackflyS USB3 camera (BFS-U3-04S2C0C, Flir, Wilsonville, OR, USA). The location of the novel object was randomized between animals. Trials in which the total exploration time of both objects lasted <5 s were considered insufficient and removed from the analysis (HI VEH *n* = 1) [38]. Animals that did not participate (e.g., jumped out of the arena) in the first 5 min of the novel object trial were omitted from the analysis (SHAM: *n* = 8, HI VEH: *n* = 4, HI Lysoveta: *n* = 2, HI MSCs: *n* = 2, HI Lysoveta + MSCs: *n* = 2). Time spent exploring the objects during the NORT, i.e., orienting the nose toward the object with a 1–2 cm distance, was scored manually by an experienced observer. Novel object preference was calculated as [(time spent with novel object/total time spent with both objects) × 100%].

#### 2.5.3. Cylinder Rearing Task

Unilateral sensorimotor impairments were measured in the cylinder rearing task on P37. Mice were placed in a transparent Plexiglas cylinder (80 mm diameter and 300 mm height) and video-recorded for 5 min. Mice were omitted from the study when they failed to perform at least 10 rearings in 5 min (SHAM: *n* = 1, HI Lysoveta: *n* = 1, HI MSCs: *n* = 2, HI Lysoveta + MSCs: *n* = 3). The first weight-bearing forepaw contacting the cylinder wall during a full rear was scored by an experienced observer as left (impaired), right (unimpaired), or both using Noldus Observer XT16.0 software (Noldus). Non-impaired forepaw preference was calculated as ((right rearings − left rearings)/(right + left + both rearings)) × 100%.

### 2.6. SH-SY5Y Culture

Human neuroblastoma cell line SH-SY5Y (#CRL-2266, ATCC, Manassas, VA, USA) was cultured in DMEM/F12 medium (#11330057, ThermoFisher) supplemented with 10% FCS (Fisher Scientific) and 1% penicillin/streptomycin (P/S, Fisher Scientific). Cells were grown in T75 flasks (Corning) in a humidified incubator (MCO-19M-PE, PHCbi, Etten-Leur, The Netherlands) with a 5% CO_2_ level at 37 °C. SH-SY5Y cells were harvested when 90% confluent (Passage 7–14) by using trypsin-EDTA (#25300-054, Invitrogen) for 2 min. Cells were centrifuged for 5 min at 300 rpm at room temperature (RT) to be collected for passaging or plating. SH-SY5Y cells were either hit with oxygen glucose deprivation (OGD), H_2_O_2_, or etoposide to examine the specific neuroprotective effects of DHA and Lysoveta. The Lysoveta product was diluted based on the DHA concentration in the product to a 1 mM stock in 0.7% DMSO (#D1370.0100, Duchefa, Haarlem, The Netherlands) in PBS, and DHA was dissolved to a 1 mM stock in 1% BSA (Merck KgA) in PBS.

#### 2.6.1. Oxygen Glucose Deprivation Model

SH-SY5Y cells were plated in a 96-well plate (Thermo Fisher Scientific) at 60,000 cells per well and were allowed to attach overnight. The next day, the culture medium was replaced with DMEM without glucose (#11966025, Merck KgA) supplemented with 1% P/S and 0 µM, 5 µM, or 10 µM Lysoveta or algae-derived DHA (#D2534, Merck KgA). Cells were incubated in a humidified hypoxic incubator with 1% O_2_ and 5% CO_2_ at 37 °C (Multigas CO_2_/O_2_ Incubator MCO-170MP-PA, PHCbi) for 24 h. In parallel, a non-OGD control plate was incubated with a culture medium containing DMSO or BSA as a vehicle in a humidified normoxic incubator with 21% O_2_ and 5% CO_2_ at 37 °C for 24 h. Neuronal cell death was assessed with a 3-[4,5-dimethylthiazol-2-yl]-2,5 diphenyl tetrazolium bromide (MTT) assay (see Section 2.6.4).

#### 2.6.2. Reactive Oxygen Species (ROS) Model: H_2_O_2_

SH-SY5Y cells were plated in a 96-well plate at 60,000 cells per well and were allowed to attach overnight. The next day, cells were exposed to 60 µM H_2_O_2_ or control medium (0 µM H_2_O_2_) in combination with 0 µM, 5 µM, or 10 µM Lysoveta or DHA in a humidified incubator with 5% CO_2_ at 37 °C for 24 h. Control conditions contained DMSO or BSA as a vehicle. Neuronal cell death was assessed with an MTT assay (see Section 2.6.4).

#### 2.6.3. DNA Damage Model: Etoposide

SH-SY5Y cells were plated in a 96-well plate at 60,000 cells per well and were allowed to attach overnight. The next day, cells were exposed to 2.8 µM etoposide (#E1383, Merck KGgA) or control medium in combination with 0 µM, 5 µM, or 10 µM Lysoveta or DHA in a humidified incubator with 5% CO_2_ at 37 °C for 24 h. Etoposide is a topoisomerase II inhibitor that induces p53-dependent cell death [39]. Control conditions contained DMSO or BSA as a vehicle. Neuronal cell death was assessed with an MTT assay (see Section 2.6.4).

#### 2.6.4. Methylthiazolyldiphenyl-Tetrazolium Bromide (MTT) Assay

After exposure to the respective hit, the medium was carefully replaced with a culture medium containing 0.5 mg/mL MTT (#M2128, Merck KGgA). MTT solution was incubated for 3 h in a humidified environment with 5% CO_2_ at 37 °C. After incubation, the medium was carefully removed from the wells and MTT crystals were dissolved in 100 µL DMSO (#23500260, VWR). Optical density was measured at 570 nm using a spectrophotometer (ThermoFisher, Multiskan GO). Background absorbance was subtracted from each well. All data points were depicted as relative values compared to the average of the negative control condition, which was not exposed to the respective hit.

### 2.7. Statistical Analysis

All data were acquired in a blinded manner. Statistical analysis was performed using GraphPad Prism 10 (GraphPad Software, Boston, MA, USA). Outliers were identified by the ROUT analysis (Q = 1%). Data were checked for normal (Gaussian) distribution using the Shapiro–Wilk normality test. If data were normally distributed, statistical analysis was performed by a comparison of more than 2 groups by a one-way ANOVA with Holm–Šidák post hoc tests. For the statistical analysis of in vitro data, one-way ANOVA with Holm–Šidák post hoc tests and a one-way ANOVA-based test for trend was performed. Statistical analysis of body weight was performed with a two-way ANOVA considering treatment and postnatal day as independent variables. If data were not normally distributed, statistical analysis was performed by a non-parametrical test with Dunn’s post hoc tests. The experimental unit was an animal or cell culture well. Data are presented as mean ± SEM. Differences of *p* ≤ 0.05 were considered statistically significant.

## 3. Results

### 3.1. Oral Lysoveta Supplementation Reduces HI Brain Injury without Affecting Body Weight

#### 3.1.1. Lysoveta Supplementation Does Not Affect Body Weight of HI-Injured Animals

**Figure 1:**
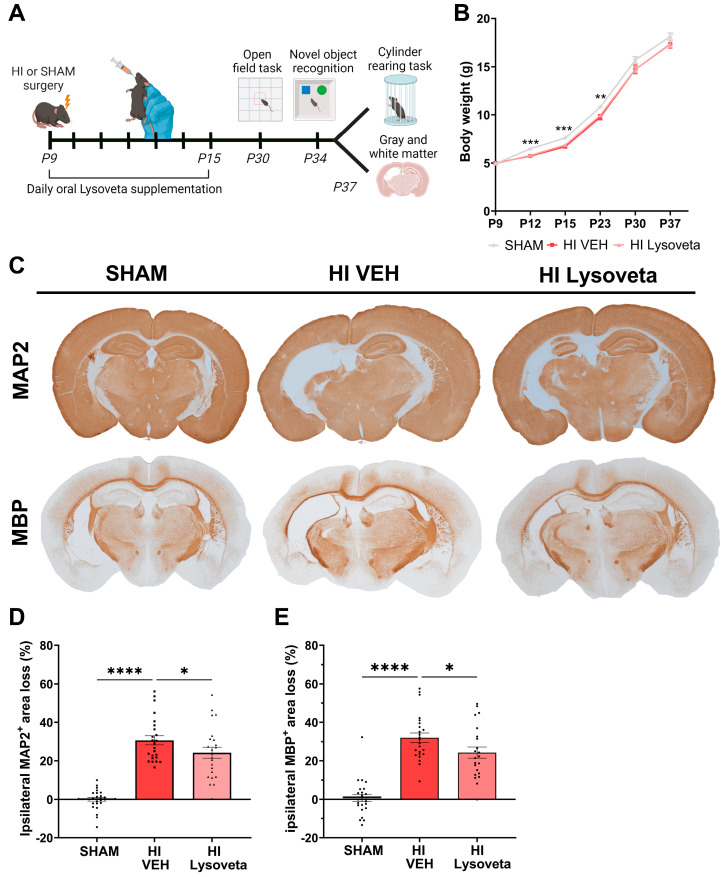
Lysoveta supplementation reduces gray and white matter loss in HI-injured animals. (**A**) Experimental setup; P: postnatal day. (**B**) Body weight during the experiment in grams of SHAM, HI-injured vehicle-treated (VEH), and HI-injured Lysoveta-treated animals, * indicates significant difference between both HI groups and SHAM animals. (**C**) Representative images of MAP2 (gray matter) and MBP (white matter) staining (in brown) in SHAM, HI-injured vehicle-treated, and HI-injured Lysoveta-treated animals. (**D**) Quantification of the ipsilateral MAP2^+^ area loss; SHAM: *n* = 24, HI VEH: *n* = 24, HI Lysoveta: *n* = 23. (**E**) Quantification of the ipsilateral MBP^+^ area loss; SHAM: *n* = 24, HI VEH: *n* = 24, HI Lysoveta: *n* = 22. * *p* < 0.05, ** *p* < 0.01, *** *p* < 0.001, **** *p* < 0.0001. Data are presented as mean + SEM.

To assess whether Lysoveta supplementation exerts neuroprotective effects following neonatal HI brain injury, Lysoveta was orally supplemented daily from P9 until P15 and the body weight of mice was monitored throughout the study (Figure 1A). Mouse pups exposed to HI showed a significantly reduced body weight compared to SHAM mice (main effect: *p* = 0.0096, Figure 1B). Post hoc comparisons revealed that differences in body weight were found between HI animals and SHAM animals from P12 until P23 (P12: *p* < 0.0001, P15: *p* < 0.0001, and P23: *p* = 0.0054). No differences in body weight were found between vehicle-treated and Lysoveta-treated HI mice, indicating no metabolic effects of Lysoveta supplementation compared to vehicle treatment.

#### 3.1.2. Lysoveta Supplementation Reduces Gray and White Matter Loss in HI-Injured Animals

HI animals orally supplemented with vehicle displayed significant MAP2^+^ area loss in the ipsilateral hemisphere compared to SHAM animals (*p* < 0.0001, Figure 1C,D), indicating gray matter injury following HI. Lysoveta supplementation significantly decreased ipsilateral MAP2^+^ area loss in HI animals compared to vehicle treatment (*p* = 0.0444). Similarly, significant MBP^+^ area loss in the ipsilateral hemisphere was observed in HI-injured animals compared to SHAM animals (*p* < 0.0001, Figure 1C,E), indicating white matter injury following HI. Lysoveta treatment significantly reduced ipsilateral MBP^+^ area loss in HI-injured animals compared to vehicle treatment (*p* = 0.0299). Therefore, Lysoveta supplementation for seven days following HI demonstrated neuroprotective effects, as evidenced by a reduction in gray and white matter loss, without being confounded by general caloric effects.

### 3.2. Oral Lysoveta Supplementation Does Not Improve Functional Outcomes after HI Injury

To assess the effects of Lysoveta supplementation on functional outcomes, at 3–4 weeks after HI induction, mice were subjected to different behavioral tests to assess anxiety-like behavior, spatial memory, and sensorimotor behavior (Figure 1A).

#### 3.2.1. Lysoveta Supplementation Does Not Reduce Anxiety-Like Behavior after HI Injury

**Figure 2:**
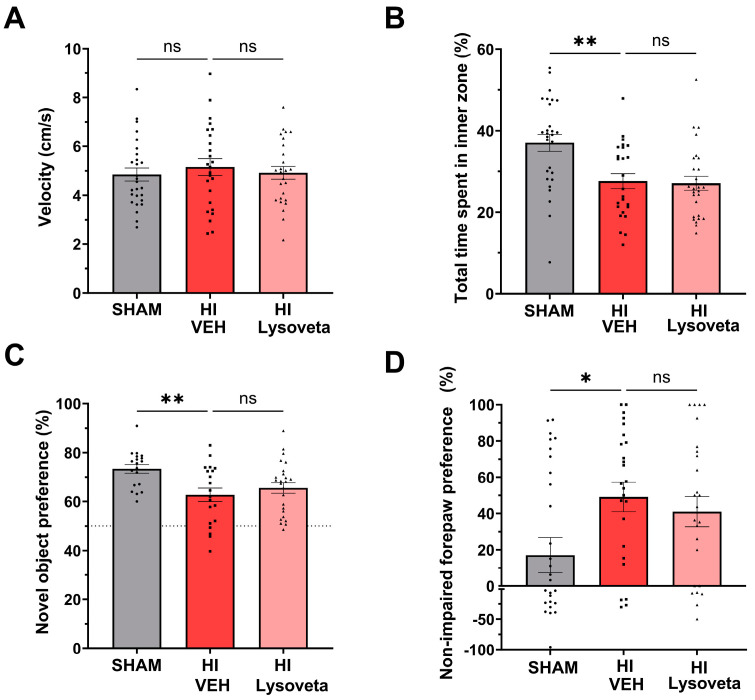
Oral supplementation with Lysoveta does not improve anxiety-like behavior, spatial memory, and sensorimotor impairments in HI-injured mice. (**A**) Quantification of the average velocity in cm/second in the open field of SHAM-operated animals and HI-injured animals treated with vehicle (VEH) or Lysoveta; SHAM: *n* = 27, HI VEH: *n* = 25, HI Lysoveta: *n* = 26. (**B**) Quantification of the total time spent in the inner zone of the open field arena; SHAM: *n* = 28, HI VEH: *n* = 25, HI Lysoveta: *n* = 27. (**C**) Quantification of the novel object preference of SHAM-operated animals and HI-injured animals treated with vehicle or Lysoveta. Dotted line at 50% chance level; SHAM: *n* = 19, HI VEH: *n* = 20 HI Lysoveta: *n* = 25. (**D**) Quantification of the non-impaired forepaw preference of SHAM-operated animals and HI-injured animals treated with vehicle or Lysoveta in the cylinder rearing task; SHAM: *n* = 27, HI VEH: *n* = 25, HI Lysoveta: *n* = 26. * *p* < 0.05, ** *p* < 0.01, ns = no significant difference. Data are presented as mean + SEM.

In the open field task, anxiety-like behavior and locomotor activity were assessed. HI-injured animals had similar movement velocities as SHAM animals, indicating that general locomotor activity was not affected by HI (SHAM vs. vehicle-treatment: *p* = 0.9840, Figure 2A). HI animals orally supplemented with the vehicle displayed anxiety-like behavior as they spent significantly less time in the inner zone of the arena than SHAM mice (*p* = 0.0032, Figure 2B). Oral Lysoveta supplementation did not improve the anxiety-like behavior of HI-injured animals compared to vehicle treatment (*p* > 0.9999).

#### 3.2.2. Lysoveta Supplementation Does Not Ameliorate Spatial Memory Impairment after HI Injury

The novel object recognition task was performed to assess spatial memory. As expected, SHAM animals spent more time (72%) with the novel object than with the familiar object (Figure 2C). HI-injured animals showed significantly reduced preference for the novel object, indicating impaired spatial memory (SHAM vs. HI vehicle-treatment: *p* = 0.0073). No benefit of Lysoveta supplementation on spatial memory functioning was found in HI-injured animals compared to vehicle treatment (*p* = 0.3798).

#### 3.2.3. Lysoveta Supplementation Does Not Reduce Sensorimotor Impairment after HI Injury

Unilateral sensorimotor impairment was assessed in the cylinder rearing task. HI-injured animals showed a significantly higher preference of using their non-impaired forepaw compared to SHAM animals (*p* = 0.0120, Figure 2D), indicating sensorimotor impairment following HI injury. Similar impairment was found in HI animals that were supplemented with Lysoveta (*p* = 0.8593 versus HI-vehicle).

### 3.3. Lysoveta Supplementation Protects Neurons against Oxygen Glucose Deprivation by Reducing Injury from Oxidative Stress

**Figure 3:**
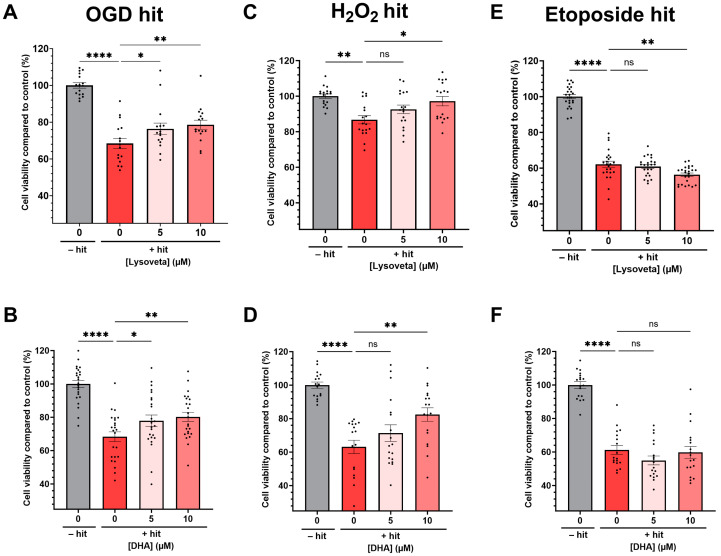
Lysoveta and DHA treatment partially protect SH-SY-5Y cells against oxygen glucose deprivation (OGD) and oxidative stress. (**A**) Quantification of the relative cell viability of SH-SY-5Y cells exposed to oxygen glucose deprivation (OGD) with 0, 5, or 10 µM (LPC-bound) DHA in Lysoveta compared to non-OGD control condition; this experiment was performed 8-fold and repeated twice. (**B**) Quantification of the relative cell viability of SH-SY-5Y cells exposed to oxygen glucose deprivation (OGD) with 0, 5, or 10 µM DHA compared to non-OGD control condition; this experiment was performed 8-fold and repeated three times. (**C**) Quantification of the relative cell viability of SH-SY-5Y cells exposed to 60 µM H_2_O_2_ with 0, 5, or 10 µM (LPC-bound) DHA in Lysoveta compared to no H_2_O_2_ control condition; this experiment was performed 6-fold and repeated three times. (**D**) Quantification of the relative cell viability of SH-SY-5Y cells exposed to 60 µM H_2_O_2_ with 0, 5, or 10 µM DHA compared to no H_2_O_2_ control condition; this experiment was performed 6-fold and repeated three times. (**E**) Quantification of the relative cell viability of SH-SY-5Y cells after exposure to 2.8 µM etoposide with 0, 5, or 10 µM (LPC-bound) DHA in Lysoveta compared to no-etoposide control condition; this experiment was performed 6-fold and repeated three times. (**F**) Quantification of the relative cell viability of SH-SY-5Y cells after exposure to 2.8 µM etoposide with 0, 5, or 10 µM DHA compared to no-etoposide control condition; this experiment was performed 6-fold and repeated three times. * *p* < 0.05 ** *p* < 0.01, **** *p* < 0.0001, ns: no significant difference. Data are presented as mean + SEM.

To explain how Lysoveta exerts neuroprotective effects in vivo, we assessed the effects of Lysoveta on different types of neuronal injury in vitro. Firstly, oxygen glucose deprivation (OGD) significantly reduced cell viability by approximately 32% compared to control (non-OGD) cells (*p* < 0.0001, Figure 3A). The addition of Lysoveta at a concentration of 5 or 10 µM (LPC-bound) DHA dose-dependently (trend analysis: *p* = 0.0142) partially protected neurons against OGD-induced cell death compared to vehicle treatment (5 µM: *p* = 0.0182, 10 µM: *p* = 0.0057). At similar dosages, DHA alone also improved cell viability dose-dependently (trend analysis: *p* = 0.0072) after OGD compared to vehicle treatment (5 µM: *p* = 0.0195, 10 µM: *p* = 0.0082, Figure 3B). To further examine whether Lysoveta protects neurons against oxidative stress or DNA damage, neuronal cultures were exposed to H_2_O_2,_ or etoposide, respectively. An oxidative stress hit with H_2_O_2_ significantly reduced cell viability compared to 0 µM H_2_O_2_ (*p* = 0.0017, Figure 3C). The addition of Lysoveta dose-dependently protected against H_2_O_2_-induced cell death (trend analysis: *p* = 0.0040) and at a concentration containing 10 µM (LPC-bound) DHA, it significantly protected neurons against H_2_O_2_-induced cell death compared to vehicle treatment (*p* = 0.0131). Similarly, DHA dose-dependently protected against H_2_O_2_-induced cell death (trend analysis: *p* = 0.0029) and 10 µM DHA significantly protected neurons against oxidative stress (*p* = 0.0017, Figure 3D). Etoposide significantly reduced cell viability compared to non-exposed cells (*p* < 0.0001, Figure 3E). Lysoveta at a dose of 5 µM (LPC-bound) DHA did not protect neurons against DNA damage induced by etoposide (*p* = 0.4635) and 10 µM Lysoveta significantly worsened cell viability compared to the vehicle-treated condition (*p* = 0.0032). Additionally, DHA alone did not protect neurons against an etoposide hit similarly to Lysoveta (Figure 3F). Conclusively, Lysoveta protected neurons specifically in an oxygen glucose deprivation model and against oxidative stress in a H_2_O_2_ hit model but did not protect neurons against DNA damage-induced cell death. As DHA alone evoked similar patterns of neuroprotection, DHA is likely partially responsible for the neuroprotective effects of Lysoveta against oxidative stress injury.

### 3.4. The Combination of Oral Lysoveta Supplementation and Intranasal MSC Therapy Does Not Improve Gray or White Matter Loss Compared to MSC Therapy Alone

**Figure 4:**
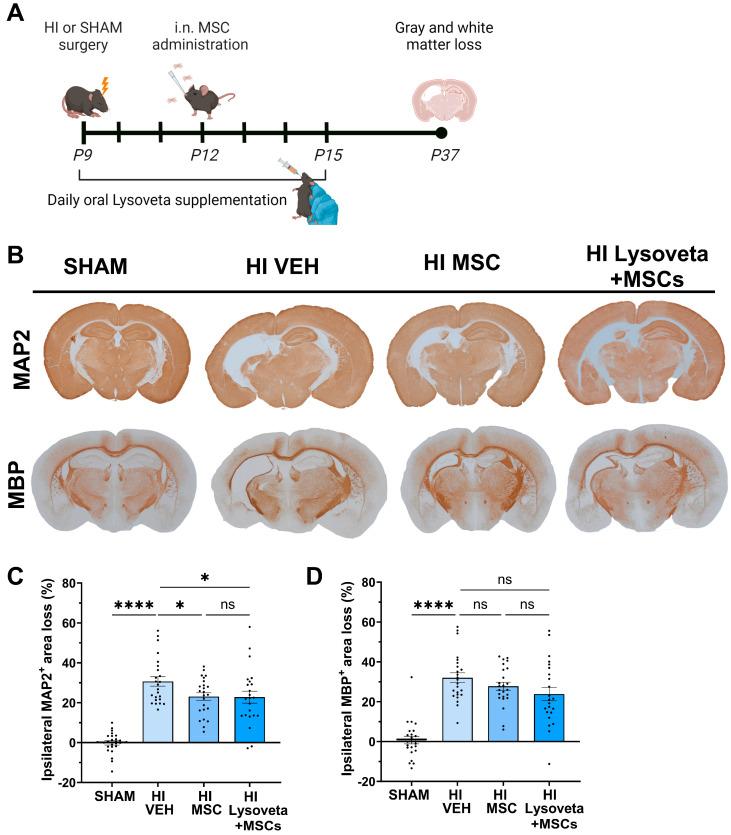
Lysoveta supplementation does not enhance the therapeutic potential of intranasal MSC therapy. (**A**) Experimental setup. i.n.: intranasal. P: postnatal day. (**B**) Representative images of MAP2 (gray matter) staining and MBP (white matter) staining in SHAM-operated animals, and HI-injured animals treated with vehicle (VEH), intranasal MSCs, or both oral Lysoveta and intranasal MSCs. (**C**) Quantification of the ipsilateral MAP2^+^ area loss; SHAM: *n* = 24, HI VEH: *n* = 24, HI MSC: *n* = 24, HI Lysoveta + MSCs: *n* = 23. (**D**) Quantification of the ipsilateral MBP^+^ area loss; SHAM: *n* = 24, HI VEH: *n* = 24, HI MSC: *n* = 24, HI Lysoveta + MSCs: *n* = 23. * *p* < 0.05, **** *p* < 0.0001, ns: no significant difference. Data are presented as mean + SEM.

To assess the potency of oral Lysoveta to enhance the regenerative efficacy of intranasal MSC therapy, HI-injured mice were treated with single or combination therapies and the anatomical outcome was assessed by measuring ipsilateral gray and white matter loss (Figure 4A).

HI-injured animals showed significant gray and white matter loss in the ipsilateral hemisphere compared to SHAM animals (*p* < 0.0001, Figure 4B–D). Intranasal MSC therapy alone and the combination therapy of MSCs and Lysoveta supplementation significantly reduced MAP2^+^ area loss in HI-injured animals compared to vehicle treatment (*p* = 0.0372) (Figure 4C). However, Lysoveta supplementation did not further reduce gray matter lesion size in MSC-treated animals compared to MSC therapy alone (*p* = 0.9306). Both intranasal MSC therapy alone and combination therapy with Lysoveta supplementation did not significantly reduce MBP^+^ area loss in HI-injured animals compared to vehicle treatment (VEH vs. MSCs: *p* = 0.4218, VEH vs. Lysoveta + MSCs: *p* = 0.0739, Figure 4D). In sum_,_ although Lysoveta supplementation alone is effective in reducing lesion size (Figure 1), the addition of Lysoveta supplementation to MSC therapy did not increase the regenerative potential of intranasal MSC therapy to reduce lesion size following HI brain injury.

## 4. Discussion

This study was conducted to assess the efficacy of Lysoveta, a product high in LPC-DHA and LPC-EPA, in a mouse model of neonatal HI brain injury and in in vitro neuronal injury paradigms.

In accordance with our hypothesis, oral Lysoveta supplementation provided protection against neuronal damage and myelin loss, thereby reducing lesion size. Importantly, no significant weight changes were observed upon Lysoveta supplementation in HI-injured animals, indicating that therapeutic effects were likely due to specific mechanisms of Lysoveta rather than general metabolic changes related to the supplementation of energy-rich lipids.

Although Lysoveta supplementation significantly reduced lesion size following HI, no functional improvements were observed in the tested behavioral paradigms upon short-term (7 days) oral Lysoveta supplementation. When we studied the brain areas related to the behavioral tasks selectively, such as the hippocampus for the novel object recognition task and the sensorimotor cortex for the cylinder rearing task [16,40], we did not find significant anatomical improvements in the lesion in these areas separately. This indicates that the discrepancy between anatomical and functional recovery in this study may be related to Lysoveta targeting other brain areas unrelated to these behavioral tasks. Moreover, to assess sensorimotor behavior, we performed the cylinder rearing task, which is widely used in the field of HI brain injury research [41,42] but might not show subtle motor improvement in the animals treated with Lysoveta supplementation. Therefore, we suggest that future research should perform other often used motor behavioral tasks such as the rotarod task to further assess potential subtle improvements by Lysoveta supplementation [41].

Previous studies using animal models of neonatal HI brain injury showed functional improvements by intraperitoneal administration of 1 to 375 mg/kg DHA [19,20,23]. In the current study, we administered 117.5 mg/kg body weight LPC-bound DHA and 210.63 mg/kg body weight LPC-bound EPA in Lysoveta daily. Although these levels are higher than those previously found sufficient to enhance brain incorporation [13,15] and sufficient to reduce lesion size in the current study, the levels might have been insufficient to provide functional recovery also. Alternatively, the timing of intragastric LPC-*n*-3 LCPUFA administration immediately after HI may have been suboptimal for functional recovery. Blood and brain *n*-3 LCPUFA levels were shown to increase within 4–12 h after oral administration of krill oil to rats [43], indicating that dietary LPC-*n*-3 LCPUFAs in our study should have reached the brain within the established neuroprotective treatment window of 6 h post-insult, but maybe not yet in sufficient amounts [42,43]. Furthermore, a study by Huang et al. indicated that long-term, but not short-term, dietary supplementation with DHA reduced functional impairment in a spinal cord injury mouse model [44]. Combining dietary DHA supplementation with acute intravenous DHA further improved functional outcome compared to acute intravenous treatment alone [44], indicating that dietary intervention may be more effective in later repair mechanisms extending the 7 days of treatment applied in this study. Additionally, more complex supplementation of multiple nutritional components might be more effective, as not only DHA and EPA are needed for phospholipid production but also zinc and vitamin B12 may be essential to contribute to functional repair [16].

Lysoveta, rich in LPC-DHA and LPC-EPA, may have additional benefits compared to non-complexed DHA or EPA supplements. LPC-DHA and LPC-EPA have been shown to significantly increase brain DHA and EPA levels and improve memory function in adult mice compared to free DHA or TAG-DHA [13,14,15]. The enhanced uptake of LPC-DHA or LPC-EPA is mediated by the Mfsd2a transporter that selectively transports LPC esterified *n*-3 LCPUFAs across the blood–brain barrier and strongly regulates lipogenesis and proper brain development [12,45]. A study by Li et al. showed that dietary supplementation with LPC-DHA upregulated Mfsd2a expression and had a protective effect on the blood–brain barrier after HI brain injury [46]. A direct back-to-back comparison of LPC-*n*-3 LCPUFAs and free *n*-3 LCPUFA treatment in animal models of neonatal HI brain injury is needed in order to determine whether the administration of LPC-*n*-3 LCPUFAs indeed shows increased therapeutic efficacy.

Lysoveta contains both LPC-bound DHA and EPA. Recent research has stressed the importance of the EPA/DHA ratio during supplementation by suggesting that EPA and DHA compete for occupancy in the phospholipid membrane pool and exert different effects on plasma membrane biophysical structure, thereby impacting cell signaling [47]. Studies of HI injury that examined both DHA and EPA separately indicated that only DHA supplementation reduced lesion size, decreased oxidative damage, and improved neurological outcomes [21,23]. These studies indicate that DHA may be the primary contributor to the observed beneficiary effects in our study. Indeed, in this study, we showed in in vitro models of neuronal damage that DHA alone exerts similar effects as Lysoveta, suggesting that DHA is at least one of its neuroprotective components. However, it is important to note that LPC-EPA supplementation was shown to increase both EPA and DHA levels in the brain [15], indicating that both forms of LPC-bound *n*-3 LCPUFAs can enhance DHA incorporation in the brain.

Our study provides evidence suggesting that Lysoveta protects against neuronal damage by reducing oxidative stress-induced injury. During the first 24 h after the HI insult, oxidative stress is an important mediator that leads to mitochondrial degradation and eventually apoptosis [2]. As blood and brain LCPUFA levels increase within 4–12 h after oral administration of krill oil [43], Lysoveta indeed may have had direct effects on oxidative stress in vivo in the current study. Indeed, intravenous administration of DHA 3.5 h after HI has been shown to reduce lipid peroxidation biomarkers in the cortex and hippocampus of piglets [48]. Additionally, tri-DHA administration within the first hour after HI resulted in the preservation of mitochondrial Ca^2+^ buffering capacity, leading to reduced oxidative injury following HI in mice [23]. Similarly, our in vitro results indicate that Lysoveta rescues neuronal cells specifically against oxidative stress-induced hits (oxygen radicals and oxygen glucose deprivation). Whether Lysoveta also reduces oxidative stress in vivo should be assessed in future short-term follow-up experiments. Additionally, other neuroprotective mechanisms of Lysoveta should be considered, including the restoration of depleted DHA levels following HI [16] and support of neurogenesis [49,50]. Importantly, the inhibition of neuroinflammation by Lysoveta should be assessed in vivo [16] and in vitro by examining the effect of Lysoveta on challenged astrocytes or microglia [51].

This study also assessed the potential additive benefit of Lysoveta supplementation on the efficacy of intranasal MSC therapy for the treatment of neonatal HI brain injury. Consistent with previous work, MSCs alone reduced neuronal tissue loss in HI-injured mice [35]. However, inconsistent with previous work, myelin tissue loss was not reduced by MSCs alone [35], which could be due to oral gavage with vehicle solution coconut oil that contains oleic acid (highly present in the myelin sheet [52]) or a gavage-induced stress response that may interfere with the regenerative effect of MSCs [53]. The results of the current study did not support our hypothesis that Lysoveta supplementation would enhance the therapeutic efficacy of MSC therapy. Upon intranasal administration, MSCs respond to chemotactic signals released in the HI lesioned tissue [24,54,55]. Possibly, Lysoveta supplementation alters the HI brain milieu to such an extent that MSCs might not be able to optimally migrate into the brain, potentially hampering additive benefits of early Lysoveta supplementation on MSC therapy [30]. Moreover, the treatment duration of Lysoveta may need to be prolonged to truly target repair and neurogenesis and complement the regenerative effects of MSC therapy [56]. Another potential opportunity to enhance MSC efficacy is by preconditioning MSCs with *n*-3 LCPUFAs prior to administration. Preconditioning MSCs with DHA has been shown to alter the content of the MSC secretome (e.g., containing oxylipins) and thereby impact the immunomodulatory properties of the MSCs [57,58]. The preconditioning of MSCs with EPA enhances the pro-resolution and anti-inflammatory effects in a mouse model of allergic asthma [59]. Conclusively, to maximize the effects of Lysoveta supplementation on MSC therapy in future studies, emphasis should be put on optimizing the timing of Lysoveta supplementation and exploring new methods to combine MSCs and the Lysoveta supplement.

## 5. Conclusions

In conclusion, our current investigation highlights the potential of early-life nutritional supplementation with LPC-bound *n*-3 LCPUFA from Lysoveta as a promising strategy to reduce neonatal HI brain injury. Short-term Lysoveta supplementation protects against gray and white matter injury but does not ameliorate the investigated functional deficits later in life in this neonatal HI animal model. The protective effects may be attributed to the antioxidant effects of the Lysoveta supplement. Lysoveta supplementation at the dose and timing used in this study did not enhance the treatment efficacy of intranasal MSC therapy. Future preclinical studies should optimize the treatment dose, timing, and duration of LPC-DHA and LPC-EPA with the aim to obtain the most beneficial treatment regime for the improvement in both anatomical and functional outcomes following neonatal HI brain injury.

## Figures and Tables

**Table 1:** Number of animals used in this study per experimental group.

Experimental Group			Number of Animals Histological Outcome			Number of Animals Behavioral Outcome		
Experimental Injury	Oral Treatment	Intranasal Treatment	Males	Females	Total	Males	Females	Total
SHAM (no HI injury)	-	-	12	12	24	14	14	28
HI injury	Vehicle	Vehicle	15	9	24	16	10	26
HI injury	Lysoveta	Vehicle	14	9	23	16	11	27
HI injury	Vehicle	MSCs	15	9	24	16	10	26
HI injury	Lysoveta	MSCs	13	11	24	15	13	28
Total number of animals used			69	50	119	77	58	135
Number of litters used					20			20

**Table 2:** Fatty acid profile of the nutritional supplementation product, Lysoveta, used in this study.

Fatty Acid Profile Lysoveta Nutritional Supplement	Content in g/100 g
Saturated fatty acids	2.40
Palmitic acid (C16:0)	1.47
Stearic acid (C18:0)	0.75
Myristic acid (C14:0)	0.18
Monounsaturated fatty acids	2.23
Oleic acid (C18:1n9)	1.45
Vaccenic acid (C18:1n7)	0.37
Palmitoleic acid (C16:1)	0.34
Cetoleic acid (C20:1n9)	0.07
*n* -3 Polyunsaturated fatty acids	32.39
Eicosapentaenoic acid (C20:5*n*-3)	16.82
Docosahexaenoic acid (C22:6*n*-3)	9.29
Stearidonic acid (C18:4*n*-3)	3.08
α-Linolenic acid (C18:3*n*-3)	1.83
Heneicosapentaenoic acid (C21:5*n*-3)	0.72
Docosapentaenoic acid (C22:5*n*-3)	0.35
Eicosatetraenoic acid (C20:4*n*-3)	0.30
*n* -6 Polyunsaturated fatty acids	0.74
Linoleic acid (C18:2*n*-6)	0.59
Arachidonic acid (C20:4*n*-6)	0.15
Total LPC	41.7
LPC-*n*-3 LCPUFA	35.8
LPC-Eicosapentaenoic acid (C20:5*n*-3)	19.3
LPC-Docosahexaenoic acid (C22:6*n*-3)	9.5

**Table 3:** Fatty acid profile of coconut oil used as a vehicle in this study.

Fatty Acid Profile Coconut Oil	Content in g/100 g
Saturated fatty acids	91.2
Lauric acid (C12:0)	45.4
Myristic acid (C14:0)	18.0
Palmitic acid (C16:0)	10.5
Capric acid (C10:0)	8.4
Caprylic acid (C8:0)	5.4
Stearic acid (C18:0)	2.3
Caproic acid (C6:0)	0.8
Arachidic acid (C20:0)	0.4
Monounsaturated fatty acids	7.9
Oleic acid (C18:1n9)	7.5
Palmitoleic acid (C16:1)	0.4

## Data Availability

The original contributions presented in the study are included in the article. Further inquiries can be directed to the corresponding author (C.G.M.d.T.).
